# Intelligence

**Published:** 1833-05

**Authors:** 


### INTELLIGENCE.

### THE EPIDEMIC INFLUENZA.

During nearly the whole of this month, a severe catarrhal epidemic has very generally prevailed in London, and many other parts of the country. But few persons have altogether escaped its influence, although in many the symptoms have been so mild as to require for their relief and removal little more than the ordinary domestic remedies. In those, however, who had suffered previously from pulmonary complaints, the epidemic has in many instances been very severe, and not unfrequently fatal. Several cases have fallen under our own care, in which the patients, who had before been subject to bronchitis and asthmatic diseases, have been placed in danger by the attack of the prevailing malady. In the course of the last fortnight we have seen upwards of 150 cases; and, if we are to credit the reports we have heard, this is but a small number in comparison with that which has fallen to the share of many other practitioners. The disease has usually commenced with headach and a general feeling of oppression; febrile symptoms of greater or less severity, modified of course by the previous state and constitution of the patient, has quickly followed; cough, hoarseness, and soreness of the throat, with sometimes slight inflammation of the eyes, have either at first attended the febrile accession, or speedily succeeded it. In many instances the patient has complained of great lassitude, and a feeling of general soreness over the whole surface of the body, with pains in the arms and legs. The cough has generally been violent and tedious.

As far as our own observation extends, children have been much less subject to the disease than adults, and females much more so than males.

We are not aware that it has proved fatal in any case where the patient was before the attack in good health. We know that many fatal cases have been mentioned, but we have found, upon inquiry, that in all these instances the patients were invalids at the period of their being attacked with the epidemic, or far advanced in age, and likely to fall victims to any disturbance of their feeble and worn-out constitutions.

It has of course been found necessary to vary the treatment according to the age and constitution of the patient. With very few exceptions, however, general bleeding has not been required, and, when it has been unnecessarily employed, great depression has followed, and but little alleviation of the cough and oppression of the chest has been obtained by it. Mild purgatives, saline diaphcretics, occasional warm drinks, and confinement to bed for a few days, has appeared to be the most effectual and beneficial mode of treatment. An opiate at bedtime has generally been given with advantage. The disease is now on the decline, but in many persons who have been attacked the harassing cough continues, and appears to be but little under the control of ordinary remedies.

Many persons, who have imprudently exposed themselves to cold soon after the attack, have suffered from severe relapses, the management of which we have found much more difficult than the original disease.

---

London Medical Society Prizes. The following subjects are fixed upon; essays to be sent on or before the 1st December in each year: 1834, Diseases of the Urethra; 1835, Pathology and Treatment of Puerperal Fever.

The Jacksonian Prize for 1832, offered for the best essay on the Mode of Union in Simple and Compound Fractures, was awarded by the Council of the College of Surgeons, to Benjamin Phillips, Esq., of Wimpole street, Cavendish square.

At a meeting of the Council of the Medico-Botanical Society of London, held 12th February, 1833, Earl Stanhope, President, in the chair,

It was resolved, That the Gold Medal of the Society shall be offered for the best Essay in the English, French, German, or Latin language, on the question, "What is the vegetable substance which could be employed with success as a substitute for mercury in the cure of Syphilis, or of diseases of the Liver?" and that the Silver Medal of the Society shall be offered for the best Essay "On the medicinal qualities and uses of any indigenous plant which is not yet sufficiently known, or on new uses and applications of any other indigenous plants," provided that such Essay possesses sufficient merit; and that they shall be received till the close of the year 1834; and that the medals shall be bestowed at the anniversary, January 16th, 1835.

That each essay shall be accompanied by a sealed paper, containing the name and address of the author, and marked in the same manner as the essay; and that each essay to which a medal is not awarded shall, according to the wish of the author, be restored to him, or submitted to the Council, in order to its being read at a general meeting.

By order of the Council,

George Glendinning, Secretary.

---

Professor Burnett will deliver a course of Lectures on Botany, at the Royal Institution of Great Britain, commencing on Tuesday the 21st of May. The following is an outline of the series.

§. Vegetable Metamorphosis.

I. Epigenesis Plantarum, or Transitional Metamorphosis, including the regular and ordinary Evolutions of Structure.

May 21st. *The four ages of plants.* The seed, the herb, the flower-bud, and the fruit of vegetables; analogous to the egg, the larvæ, the chrysalis, and the imago states of insects. These epochs often marked by notable changes of form, character, and function.

May 28th. *Accommodation to external physical influences.* Acclimation, forcing, unseasonable development. Apparent perceptivity in vegetables.

II. Prolepsis Plantarum, or Metastatic Metamorphosis; including the irregular and compensating developments.

June 4th. *Abnormal evolutions of root and stem.* Aërial roots; fasciculate and spiral stems; anomalous branches; foliar construction of stems and boughs; abortive buds and branches; thorns, spinacules, &c.

June 11th. *Abnormal development of buds.* Adventitious germs; arrest of development in the foliage forming the scales of the hybernacle, phyllodia, tendrils, &c., and causing the relapse of bracteæ, sepals, petals, stamens, pistils, and fruit, into leaves.

III. Anamorphosis Plantarum, or Adventitious Metamorphosis; including extraordinary and casual Transformations.

June 18th. *Changes wrought in plants by chance and culture.* Preternatural development and abortion of roots, stems, buds, leaf-stalks, leaves, flower-stalks, flowers, fruits, and seeds; hypertrophy and atrophy of these several parts.

June 25th. *Limits of the transformations observable in plants.* Appetency for improvement; no tendency to absolute change; difficulty of preserving casual varieties. Are new parts or organs ever formed? Are plants convertible into each other, or into animals of any grade? Doctrine of progressive perfectibility considered.

---

NEW ZEALAND HEADS.

It may not be generally known that the importation of New Zealander's heads is now entirely prohibited. By the late Act, which regulated the duties to be levied on anatomical preparations, an express provision is made, that not any duty, however high, shall permit them to pass; for, revolting as the statement may appear, these chiefs were absolutely being murdered to supply the cabinets of European virtuosi; and so profitable had the trade become, and so great was the demand, that no New Zealander's head was safe upon his shoulders, if within the reach of a trader's swivel.

Since writing the above, we have noticed the following paragraph, which has been copied into our journals from the "Sydney Herald."

"*Dangerou Cargo*. A very severe encounter took place, a short time ago, betwixt one of our colonial schooners, and a party of New Zealanders. It appears that this vessel, during her last trip to Sydney, had procured a number of native heads, and also brought up a chief with them. While the chief was on-board, the captain had occasion to overhaul his stock of heads, and they were incautiously exposed to the view of his companion. The chief caught a glimpse of them, and recognized the heads of some of his own relatives. He said but little at the time, but, when he returned to the islands, he procured a party to resent the insult given him. They immediately commenced firing upon the schooner, and a regular fire was kept up between both parties for some time, when the vessel weighed anchor, and sheered off. It is more than probable that, if the schooner had not crowded all sail, the captain and crew would have been sacrificed, and an exchange of heads taken place."—[*Sydney Herald*, Aug. 2.]

Good

---

Baron HEURTELOUP performed the operation of Lithotripsy, on Saturday last, before a numerous assemblage of medical men and students, in the theatre of St. Bartholomew's Hospital, on a patient of Mr. EARLE. The patient was a middle-aged man; the stone was seized and crushed with the greatest ease; and all present were astonished at the Baron's dexterity.

### LITERARY INTELLIGENCE.

In the press, and speedily will be published, a Translation of the celebrated Work of RAYER on the Diseases of the Skin, by WM. DICKINSON, Esq., Surgeon.

---

### MONTHLY LIST OF MEDICAL BOOKS.

### METEOROLOGICAL REGISTER,

By Messrs. HARRIS and Co., Mathematical Instrument Makers, 50, High Holborn.

| | Ra n gauge. | Moon. | Thermometer. | | | Barometer. | | De Luc's Hygrometer | | Winds. | | Atmospheric Variation. | | |
|---|---|---|---|---|---|---|---|---|---|---|---|---|---|---|
| Mar. | | | 9 a.m. | max. | min. | 9a.m. | 10p.m. | 9a.m. | 10p.m. | 9 a.m. | 10p.m. | 9 a.m. | 2 p.m. | 10 p.m. |
| 20 | | | 36 | 43 | 34 | 29.93 | 29.83 | 77 | 77 | N | NE | fine | fine | fine |
| 21 | | ● | 39 | 42 | 35 | .71 | .69 | 75 | 74 | N | NNE | showery | | |
| 22 | .08 | | 38 | 42 | 29 | .71 | .73 | 73 | 76 | NE | E | fine | | snow |
| 23 | | | 34 | 39 | 31 | .72 | .75 | 75 | 74 | NE | E | foggy | | snow |
| 24 | | | 34 | 42 | 35 | .66 | .64 | 76 | 78 | NE | NE | cloudy | cloudy | cloudy |
| 25 | | | 38 | 43 | 34 | .63 | .59 | 75 | 76 | ENE | N | fine | fine | |
| 26 | .24 | | 37 | 42 | 35 | .65 | .78 | 77 | 77 | WSW | SSW | cloudy | | fine |
| 27 | | | 39 | 43 | 36 | .87 | .84 | 77 | 78 | SE | SE | fine | | |
| 28 | | | 40 | 49 | 39 | .81 | .81 | 78 | 76 | SSE | WSW | | | |
| 29 | | ☽ | 45 | 50 | 40 | .74 | .56 | 75 | 75 | WSW | SW | fog | | cloudy |
| 30 | | | 46 | 52 | 39 | .51 | .57 | 73 | 70 | NW | NW | fine | | fine |
| 31 | .21 | | 46 | 49 | 43 | .34 | .06 | 78 | 80 | | SE | | | cloudy |
| Apr. 1 | | | 48 | 53 | 48 | 28.91 | 28.82 | 83 | 83 | SE | WSE | cloudy | | cloudy |
| 2 | .24 | | 47 | 54 | 48 | .85 | .98 | 81 | 82 | SW | SW | fog | showery | cloudy |
| 3 | | | 51 | 56 | 46 | 29.30 | 29.45 | 82 | 78 | NW | WSW | | fine | fine |
| 4 | | ○ | 50 | 57 | 43 | .33 | .29 | 80 | 76 | SSW | SSW | showery | cloudy | rain |
| 5 | | | 48 | 55 | 45 | .64 | .71 | 74 | 71 | NW | WSW | fine | fine | fine |
| 6 | | | 50 | 55 | 43 | .76 | .73 | 70 | 67 | W | WSW | | | |
| 7 | | | 47 | 54 | 41 | .73 | .73 | 78 | 70 | N | NE | | | |
| 8 | | | 49 | 55 | 43 | .74 | .84 | 71 | 70 | NE | E | | | |
| 9 | | | 51 | 56 | 48 | .87 | .78 | 70 | 72 | SW | NW | | | cloudy |
| 10 | .38 | | 53 | 67 | 43 | .65 | .42 | 73 | 69 | W | WSW | | cloudy | rain |
| 11 | | | 48 | 55 | 40 | .19 | .12 | 71 | 71 | WNW | W | cloudy | cloudy | fine |
| 12 | .15 | ☾ | 46 | 50 | 42 | .14 | .36 | 71 | 70 | NW | N | rain | cloudy | |
| 13 | | | 47 | 51 | 39 | .54 | .40 | 69 | 68 | N | W | cloudy | fine | rain |
| 14 | | | 44 | 51 | 36 | .20 | .39 | 70 | 75 | NNW | N | | rain | cloudy |
| 15 | .32 | | 42 | 45 | 35 | .31 | .10 | 76 | 72 | W | N | | rain | rain |
| 16 | | | 45 | 49 | 36 | .21 | .34 | 72 | 72 | E | ENE | | showery | fine |
| 17 | .03 | | 44 | 48 | 40 | .36 | .38 | 72 | 73 | N | NW | fine | fine | cloudy |
| 18 | | | 45 | 48 | 39 | .46 | .56 | 73 | 72 | NW | N | | showery | fine |
| 19 | | | 47 | 52 | 43 | .77 | .83 | 71 | 72 | WNW | WSW | | cloudy | |

The quantity of Rain fallen in March, was 1 inch and 1.100th.

